# Trends in adult chlamydia and gonorrhoea prevalence, incidence and urethral discharge case reporting in Mongolia from 1995 to 2016 – estimates using the Spectrum-STI model

**DOI:** 10.5365/wpsar.2017.8.2.007

**Published:** 2017-12-01

**Authors:** Jugderjav Badrakh, Setsen Zayasaikhan, Davaalkham Jagdagsuren, Erdenetungalag Enkhbat, Narantuya Jadambaa, Sergelen Munkhbaatar, Melanie Taylor, Jane Rowley, Guy Mahiané, Eline Korenromp

**Affiliations:** a Mongolia National Center for Communicable Diseases (NCCD), AIDS/STI Surveillance and Research Department.; b World Health Organization Country Office Mongolia.; c Mongolia Global Fund-supported project on AIDS and TB.; d World Health Organization, Dept. of Reproductive Health and Research, Geneva, Switzerland.; e Centers for Disease Control and Prevention, Division of STD Prevention, Atlanta, Georgia, USA.; f London, United Kingdom.; g Avenir Health, Glastonbury, CT, USA.; h Avenir Health, Geneva, Switzerland.

## Abstract

**Objective:**

To estimate Mongolia’s prevalence and incidence trends of gonorrhoea and chlamydia in women and men 15–49 years old to inform control of STIs and HIV, a national health sector priority.

**Methods:**

We applied the Spectrum-STI estimation model, fitting data from two national population surveys (2001 and 2008) and from routine gonorrhoea screening of pregnant women in antenatal care (1997 to 2016) adjusted for diagnostic test performance, male/female differences and missing high-risk populations. Prevalence and incidence estimates were then used to assess completeness of national case reporting.

**Results:**

Gonorrhoea prevalence was estimated at 3.3% (95% confidence interval, 1.6–3.9%) in women and 2.9% (1.6–4.1%) in men in 2016; chlamydia prevalence levels were 19.5% (17.3–21.9%) and 15.6% (10.0–21.2%), respectively. Corresponding new incident cases in women and men in 2016 totalled 60 334 (36 147 to 121 933) and 76 893 (35 639 to 254 913) for gonorrhoea and 131 306 (84 232 to 254 316) and 148 162 (71 885 to 462 588) for chlamydia. Gonorrhoea and chlamydia prevalence declined by an estimated 33% and 11%, respectively from 2001 to 2016.

Comparing numbers of symptomatic and treated cases estimated by Spectrum with gonorrhoea case reports suggests that 15% of symptomatic treated gonorrhoea cases were reported in 2016; only a minority of chlamydia episodes were reported as male urethral discharge cases.

**Discussion:**

Gonorrhoea and chlamydia prevalence are estimated to have declined in Mongolia during the early 2000s, possibly associated with syndromic management in primary care facilities and improving treatment coverage since 2001 and scale up of HIV/STI prevention interventions since 2003. However, prevalence remains high with most gonorrhoea and chlamydia cases not treated or recorded in the public health system.

## Introduction

Control of sexually transmitted infections (STIs) and HIV is a health sector priority in Mongolia. Since 2001, syndromic case management is implemented in primary care facilities that lack capacity for laboratory diagnosis. Prevention services targeted at high-risk groups have been intensified since 2003 with support from the Global Fund to Fight AIDS, Tuberculosis and Malaria. (*1*) In Mongolia, laboratory-diagnosed syphilis, gonorrhoea and trichomoniasis, as well as syndromically diagnosed male urethral discharge (UD) and genital ulcer disease from health facilities not doing laboratory diagnosis, are reportable; approximately 15 000 new STI cases are registered annually. (*2*-*5*) However, the true burden is believed to be higher due to undiagnosed and untreated cases, cases treated but not reported through private-sector facilities and those self-treated through pharmacies. (*6*-*8*)

Syphilis surveillance draws on periodic serological surveys and routine, near-universal screening among pregnant women attending antenatal care (ANC). For gonorrhoea and chlamydia, however, no such systematic measurement is in place.

In 2017 Mongolia, with support from the World Health Organization (WHO) and Avenir Health, estimated its adult prevalence trends for chlamydia and gonorrhoea using the Spectrum-STI model (*9*) to inform strategic planning for its STI response and strengthen its STI surveillance system.

The Spectrum-STI tool estimates trends in adult prevalence and incidence of STIs at the national level using data from routine STI surveillance and population-based surveys. (*9*)

This article presents Spectrum estimates of adult prevalence and incidence of gonorrhoea and chlamydia in Mongolia from 1995 to 2016 using prevalence survey data. Estimated male gonorrhoea and chlamydia case numbers were compared to UD case reports to estimate treatment coverage and reporting completeness. This study represents the first national-level STI trend estimation in an Asian country using an internationally agreed approach and assumptions.

## Methods

### Overview

The Spectrum-STI tool (http://avenirhealth.org/software-spectrum.php) (*9*) estimated prevalence and incidence of gonorrhoea and chlamydia in adults aged 15−49 years. Data and assumptions were reviewed at a three-day technical workshop held in Mongolia in February 2017. Participants included representatives of the Ministry of Health, HIV/AIDS and Maternal and Child Health programmes, the central reference laboratory and partners supporting or implementing the national HIV/STI response. Mongolia-specific parameter values and results from the base-case analysis were agreed at the workshop and are summarized here. Spectrum default parameter values have been described elsewhere. (*9*)

### Prevalence estimation

National prevalence levels for adult women were estimated over time as a moving average through all data points.

For both STIs, prevalence data were identified from studies conducted between 1995 and 2016 in representative general adult populations. For Mongolia, this included pregnant women attending ANC; no prevalence data were identified from any other low-risk populations.

Prevalence data from each study were adjusted for sensitivity and specificity of diagnostic tests used (*10*-*12*) ((Supplemental Digital Content (SDC) 1)). For gonorrhoea the national data from routine screening of women attending ANC ((SDC2)) used culture or Gram stain on cervical or vaginal swabs; sensitivity of these tests was set at 35% to reflect challenges in testing in routine care settings.

Each prevalence data point was adjusted upward by 10% to account for the contribution of higher-risk populations. (*10*)

National sample surveys were assigned a weight of 100% (the maximum, given that these should be nationally representative). Routine screening data were assigned a 40% weight, as agreed at the national workshop, as these were not nationwide or systematically sampled. Since the 40% value was somewhat arbitrary, we present estimates using different weights as sensitivity analysis.

Since no prevalence data were available for men, male prevalence was inferred from female estimates by applying a time-constant male-to-female prevalence ratio of 0.86 (range 0.58–1.15) for gonorrhoea and 0.80 (range 0.53–1.07) for chlamydia with uncertainty bounds incorporating both uncertainty in female prevalence and in the male-to-female ratio. (*10*)

The 95% uncertainty or confidence intervals were generated to account for binomial sampling variability in prevalence observed in the data and modelling error. (*9*) Test-adjusted prevalence rates were simulated (in 10 000 replications) following β distributions to which we added random terms in the logit scale; the random terms were sampled from a uniform distribution on residuals obtained after fitting the original data set.

### Incidence estimation and STI episode durations

Incidence was estimated by dividing estimated prevalence by an assumed average duration of infection. (*13*) The 95% confidence intervals on incidence reflect uncertainty in both the underlying prevalence (estimated by bootstrap) and in the duration of infection set at ± 50%.

STI episode durations were as assumed in the WHO 2012 global and regional estimates. (*10*) In the WHO estimates, the region that Mongolia is a member of was assumed to have intermediate treatment coverage. (*10*) However, following discussions at the national workshop, and lacking national population-based data about STI treatment coverage, we decided to use longer STI durations, reflecting low treatment coverage.

Assuming 35% treatment coverage of symptomatic gonorrhoea and chlamydia episodes in men and 22.5% in women, (*10*, *14*) we calculated average durations of gonorrhoea and chlamydia episodes weighted between the fractions treated and untreated ((SDC3)). The average duration in men was 0.32 years for gonorrhoea and 0.86 years for chlamydia and 0.47 years for gonorrhoea and 1.22 years for chlamydia in women ((SDC3)).

### STI case reporting completeness

An expected case load for UD was estimated from Spectrum-estimated case incidence, assuming that 64% of gonorrhoea cases and 14% of chlamydia cases are symptomatic and 35% of these are treated (*10*) ((SDC3)). The Spectrum estimates of symptomatic gonorrhoea cases and UD cases were then compared to national-level case reports for laboratory-diagnosed gonorrhoea and UD (a non-overlapping set of cases without laboratory diagnosis) from 1995 to 2016 collected by the National Center for Communicable Diseases (*15*, *16*) ((SDC4)) to estimate reporting completeness.

### Sensitivity analysis

Univariate sensitivity analyses assessed the sensitivity of 2016 estimates to key assumptions and Mongolia-specific input data and assumptions: the weight of routine ANC screening data points; gonorrhoea relative to national ANC surveys; the sensitivity of culture and wet-mount in routine ANC gonorrhoea screening; the gonorrhoea prevalence data used; the decline rate in chlamydia (based on few data points) relative to that in gonorrhoea (based on a longer and more continuous time series); and (as determinant of reporting completeness) the gonorrhoea incidence rate in men 50–64 years. More general and global assumptions of the Spectrum methodology were addressed elsewhere. (*9*)

## Results

### Gonorrhoea and chlamydia prevalence

**Fig. 1:**
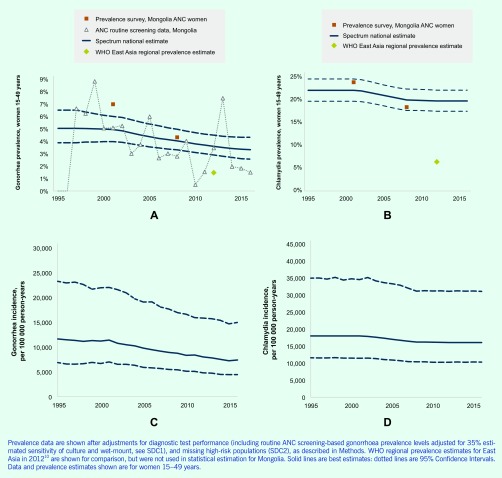
Prevalence of (A) gonorrhoea and (B) chlamydia and incidence rate of (C) gonorrhoea and (D) chlamydia in women 15–49 years in Mongolia

Two national surveys were identified from the general population; both measured gonorrhoea and chlamydia in pregnant women attending ANC in 2001 (*17*) and 2008 (*18*) ((SDC2), **Fig. 1A & B**). For gonorrhoea, national prevalence data were also available for 1997 to 2016 from routine screening of women attending ANC.

Prevalence data are shown after adjustments for diagnostic test performance (including routine ANC screening-based gonorrhoea prevalence levels adjusted for 35% estimated sensitivity of culture and wet-mount, see SDC1), and missing high-risk populations (SDC2), as described in Methods. WHO regional prevalence estimates for East Asia in 2012 (*10*) are shown for comparison, but were not used in statistical estimation for Mongolia. Solid lines are best estimates: dotted lines are 95% Confidence Intervals. Data and prevalence estimates shown are for women 15–49 years.

For gonorrhoea in women, Spectrum estimated a stable 5.0% prevalence from 1995 to 2001 followed by a decline to 3.3% (95% confidence interval, 1.6–3.9%) in 2016 (**Fig. 1A**). For chlamydia, estimated prevalence fell from 2001 (the year of the first survey) and 2008 (the year of the second and final survey), and the model thereafter assumed stable prevalence. In 2000–2001 chlamydia prevalence was 21.9% (19.5–24.4%) and in 2016 19.6% (17.3–21.9%, **Fig. 1B**). From 2001 to 2016, gonorrhoea and chlamydia prevalence declined by 33% and 11%, respectively.

No data were identified for men; therefore, male gonorrhoea and chlamydia estimates were based on female estimates (see Methods). In men, gonorrhoea prevalence was estimated at 4.3% between 1995 and 2001 and falling to 2.9% (1.6–4.1%) by 2016. For chlamydia, prevalence was estimated at 17.5% (15.6–19.5%) in 2000 and 15.6% (10.0–21.2%) in 2016.

### Gonorrhoea and chlamydia incidence

**Fig. 1C & D** show estimated trends in gonorrhoea and chlamydia incidence. In 2016, Spectrum estimated 60 334 (36 147–121 933) and 76 893 (35 639 to 254 913) new gonorrhoea cases in women and men aged 15–49 years, respectively and 131 306 (84 232 to 254 316) new cases of chlamydia in women and 148 162 (71 885 to 462 588) in men (**Table 1**). For both STIs, incidence was higher in men than in women despite higher prevalence in women, reflecting longer average duration of both infections in women than in men.

**Table 1:** Spectrum-estimated prevalence and incidence rate (per 100 000 person-years) of gonorrhoea and chlamydia in women and men 15–49 years, Mongolia in 2016

STI	Metric	Women		Men	
**-**	**-**	Point estimate	95% CI	Point estimate	95% CI
Gonorrhoea	Prevalence	3.3%	1.6–3.9%	2.9%	1.6–4.1%
	Incidence rate per 100 000 total adult population	7409	4439–14 974	9316	4318–14 314
	New incident cases, 15–49 years	60 334	36 147–121 933	76 893	35 639–254 913
Chlamydia	Prevalence	19.5%	17.3–21.9%	15.6%	10.0–21.2%
	Incidence rate per 100 000 total adult population	16 023	10 279–31 034	18 184	8823–27 546
	New incident cases, 15–49 years	131 306	84 232–254 316	148 162	71 885–462 588

95% CI = 95% confidence interval

**Fig. 2:**
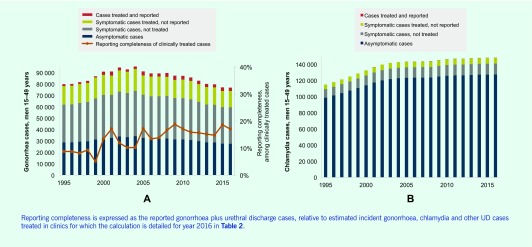
Spectrum-estimated incident gonorrhoea and chlamydia cases in men 15–49 years by treatment and reporting status, Mongolia

Gonorrhoea’s estimated case incidence rate declined in women from 11 650/100 000 in 1995 to 7409 in 2016 and in men from 13 991 to 9316 (**Fig. 1C**). Over this period, chlamydia incidence fell from 17 953 to 16 023/100 000 in women and from 20 374 to 18 184/100 000 in men (**Fig. 1D**). For chlamydia, annual incident case numbers increased slightly from 1995 to 2002, a period when prevalence (**Fig. 1B**) and incidence rates (**Fig. 1D**) were estimated to have been stable; population growth implied slightly increasing annual case numbers (**Fig. 2B**). From 2002 to 2016, annual chlamydia cases were stable (**Fig. 2B**), reflecting the counterbalancing effects of declining prevalence and incidence rates (**Fig. 1B**** and ****1D**) and population growth.

Reporting completeness is expressed as the reported gonorrhoea plus urethral discharge cases, relative to estimated incident gonorrhoea, chlamydia and other UD cases treated in clinics for which the calculation is detailed for year 2016 in Table 2.

### Gonorrhoea reporting completeness and treatment coverage

**Fig. 2A** shows Spectrum-estimated incident gonorrhoea cases in men from 1995 to 2016 split into episodes symptomatic and asymptomatic, treated and untreated and reported and unreported. Comparing national gonorrhoea case reports ((SDC4)) with Spectrum estimates of the number of men who were symptomatic and treated, reporting completeness for gonorrhoea cases treated in clinics improved from 8% in 1997 to 15% in 2016 (**Table 2**).

**Table 2:** Incident cases of gonorrhoea, chlamydia and urethral discharge and the subset who are treated and reported in men 15–49 years in Mongolia, 2016

**-**	Gonorrhoea	Chlamydia	Comment & source
New incident cases (Spectrum estimate)	76 893	148 162	Spectrum-STI estimate
Gonorrhoea or chlamydia cases developing UD/symptoms (Spectrum estimate)	49 212	20 743	Assuming 64% of male gonorrhoea (*10*) and 14% of male chlamydia cases develop symptoms (*13*, *19*) & SDC3
UD cases due to gonorrhoea or chlamydia treated in a clinic (Spectrum estimate)	17 224	7260	Assuming 35% of gonorrhoeal & chlamydial UD cases seek and get treatment (*10*) & SDC3
Actual case reports	Gonorrhoea: 2 625; UD (unknown etiology): 606		Mongolia National Center for Communicable Diseases
Gonorrhoea reporting completeness among gonorrhoea cases treated in clinics	15%		= 2 625/17 224
Reported cases relative to population-level incidence – gonorrhoea and chlamydia	1.4%		= (2 625 + 606)/(76 893 + 148 162)

UD = urethral discharge

### Chlamydia treatment and reporting coverage, 1995–2016

Of the Spectrum-estimated 148 162 chlamydia cases in men aged 15–49 years in 2016, some 20 743 were expected to be symptomatic of which 7260 would have been treated (**Table 2** and **Fig. 2B**).

Laboratory diagnosis is not commonly practiced for chlamydia and chlamydia is not reportable; chlamydia cases in men should instead be reported as UD cases per the syndromic management policy. However, reported UD cases ranged between just 342 and 1648 in 2001 and in 2005 to 2015; years 2002 to 2004 were missing.

In 2016, of population-level estimated incident gonorrhoea plus chlamydia cases (76 893 gonorrhoea plus 148 162 chlamydia; total 225 055), the reported 2625 gonorrhoea cases plus 606 UD cases covered only 1.4%. This calculation assumed all UD cases were due to gonorrhoea and/or chlamydia; in reality, not all UD cases are caused by gonorrhoea or chlamydia ((SDC5)), thus these reporting completeness estimates are optimistic. Most unreported chlamydia cases were asymptomatic (69% in 2016, **Fig. 2B**), symptomatic but not treated (20% at 2016) or treated but not reported (10% in 2016).

### Sensitivity analysis

Excluding routine ANC data from estimations, increased estimated gonorrhoea rates in 2016 (4.2% instead of 2.9% in men) and reporting completeness of male gonorrhoea cases was correspondingly lower. Conversely, when ANC routine data points were given an increased weight, namely the same weight as each ANC survey, the estimated gonorrhoea prevalence and incidence fell slightly (male prevalence 2.7% instead of 2.9%) and gonorrhoea reporting completeness increased.

Gonorrhoea results were also very responsive to the assumed sensitivity of culture in routine ANC-based screening: when varying the sensitivity between 25−75.7% (*9*) male prevalence in 2016 varied from 1.5−3.9% and gonorrhoea reporting completeness from 11–29%.

Had gonorrhoea prevalence in Mongolian men in 2016 been as low as 1.28% (the WHO 2012 estimate for men in East Asia) instead of our national estimate of 2.9%, then gonorrhoea reporting completeness would have been 34% instead of 15% completeness.

Our analysis assumed that national case report was for men 15−49 years old. In fact, some reported cases will have been from older men. Estimated gonorrhoea reporting completeness would be 13% instead of the best estimate of 15% if we assume additional gonorrhoea and chlamydia cases to occur in men above 49 years at a rate equal to men 15–49 years.

Finally, Spectrum estimates for chlamydia were based on two national surveys (2001 and 2008) and it was assumed that prevalence was constant after 2008. If chlamydia prevalence, however, fell between 2008 and 2016 at the same rate as gonorrhoea prevalence fell, the chlamydia prevalence in 2016 would have been 13% rather than 15.6%.

## Discussion

Prevalence trend estimations for Mongolia generated using the Spectrum-STI model and data from ANC-based surveys and routine screening indicate that prevalence of both chlamydia and gonorrhoea fell from 2001 to 2016. However, prevalence levels remained extremely high: for both STIs, Mongolia’s estimated prevalence in 2012 was considerably above WHO estimates for the East Asia and central Asia regions (*10*) (**Fig. 1A & B**).

Chlamydia was estimated five times more prevalent than gonorrhoea as shown in the WHO 2012 estimates globally and for Asia. (*10*) From 2001 to 2016, the estimated decline was stronger for gonorrhoea than for chlamydia, reflecting the gonorrhoea decline observed in ANC routine testing. We cannot exclude that for chlamydia the decline from 2001 to 2008 also continued after 2008 (see **Table 3**); however, there are no data post-2008 to establish this. Conversely, the estimated chlamydia decline from 2001 to 2008, based on two surveys, is our best estimate but is not as robust as a trend estimate based on multiple data points might have been. Estimates of chlamydia rates and especially their time trend are therefore less certain than for gonorrhoea.

**Table 3:** Sensitivity analysis – effect of varying (selected) assumptions and values on national estimates of gonorrhoea and chlamydia prevalence and incidence and the estimated reporting completeness for symptomatic treated gonorrhoea in Mongolian men 15–49 years, in 2016

Parameter	Default assumption	Alternative assumption		Prevalence		Incidence per 100 000		Gonorrhea case reporting completeness
		Lower	Upper	Gonorrhoea	Chlamydia	Gonorrhoea	Chlamydia	
Best/default estimate	**-**	**-**	**-**	2.9%	15.6%	9316	18 184	15.2%
Weight of routine ANC screening data points, gonorrhoea, relative to national ANC surveys	40%	0%	-	4.2%	As default	13 735	As default	10%
	-	-	100%	2.7%	As default	8724	As default	16%
Sensitivity of culture and wet-mount, in routine ANC gonorrhoea screening	35%	25%	-	3.9%	As default	12 622	As default	11%
	-	-	75.7% (*9*)	1.5%	As default	4841	As default	29%
Gonorrhoea prevalence as WHO’s East Asia regional estimate of 2012 (*10*)	Fitted to Mongolia data	-	-	1.28%	As default	4172	As default	34%
Chlamydia prevalence: decline from 2008–2016 proportional to the estimated prevalence decline over 2008–2016 in gonorrhoea	Constant from 2008–2016	-	-	As default	13.0%	As default	15 138	As default
Gonorrhoea incidence rate in men 50–64 years	0	-	Same as at 15–49 years	As default	As default	As default	As default	13% (*1*)

The declines in gonorrhoea and chlamydia prevalence are attributable to several factors including the expanded HIV/STI response and scale-up of (Global Fund-supported) HIV/STI prevention interventions including outreach services with communication, counselling and HIV and STI testing for key groups since 2003. (*1*) The declines are in line with Spectrum estimations for syphilis in ANC women. (*20*)

Declining prevalence of both gonorrhoea and chlamydia based on ANC data contrasts with stable or possibly increasing gonorrhoea and chlamydia prevalence in female sex workers (FSW) in Ulaanbaatar sampled through Integrated Bio-Behavioural Surveillance; FSW gonorrhoea prevalence increased from 13.6% in 2002 (*21*, *22*) to 15.6% in 2009 (*23*) and from 19.3% to 24.5% for chlamydia. This trend may be a true increase or reflect that in 2009 a higher-risk FSW population was sampled.

While gonorrhoea is the predominant cause of UD cases seen in clinics, (*15*, *16*) at the population level the prevalence, incidence and case numbers are much higher for chlamydia. Still large numbers of gonorrhoea and especially chlamydia cases are not treated because many infections in both men and women do not cause symptoms and because over half of symptomatic chlamydia-infected adults do not access treatment.

Comparison of reported male gonorrhoea case numbers with Spectrum-estimated incident cases suggests that in 2016 15% of male symptomatic gonorrhoea cases were treated and reported through public sector providers. Estimated reporting completeness improved after 2001 (8%), coinciding with Mongolia’s phased roll-out of syndromic STI treatment from a WHO-supported pilot in 2001 (*1*) to nationwide implementation in 2005. (*24*, *25*) In Spectrum simulations, treatment rates were assumed constant over time. If, however, treatment coverage improved then annual numbers of new cases (for a given prevalence) may have been higher and reporting completeness lower. The low reporting completeness highlights the need to strengthen Mongolia’s national reporting system and ensure it covers both public and private providers. (*8*) National surveys in 2010 and 2014 found that up to half of self-reported STI treatments were in the private sector (including purchases from pharmacies), (*14*, *26*) yet only 9% of reported cases were from private clinics in 2015. (*24*)

Despite recent declines, Mongolia has STI rates higher than neighbouring countries. This probably reflects persistent poor coverage of effective STI treatment by qualified providers. The high STI rates constitute a persistent risk factor for the possible future spread of HIV. (*27*)

### Limitations

The estimations are limited by the quality and quantity of prevalence data, modelling assumptions and assumptions made when data was lacking. (*9*) For prevalence estimation, uncertain assumptions include that all prevalence data were from ANC women who may (as for HIV) not be representative for non-ANC non-pregnant women. (*28*) Uncertainties particularly affected results for men (based on female estimates, applying a global fixed male-to-female prevalence ratio) and results for chlamydia (with less national data than gonorrhoea).

Incidence estimates depended on treatment coverage and assumed durations of treated and untreated infections for which longitudinal data are lacking. Assumed proportions of episodes that become symptomatic were calibrated on WHO estimates for the East Asia region (*10*) not on Mongolia-specific data. Proportions of symptomatic episodes that get treated were also taken from regional-level WHO assumptions (*10*) where we situated Mongolia as a country with low treatment coverage, an assumption we could not validate against population-based local data.

Finally, the assessment of gonorrhoea and UD case reporting completeness required additional assumptions, most of which were global rather than Mongolia-specific.

### Implications for surveillance and programmatic response

The last national STI survey in Mongolia was conducted in 2008 in ANC women, and as of 2017 no survey has measured STI prevalence levels in low-risk men. Mongolia would benefit from regular population-based prevalence measurements looking at multiple STIs in low-risk men and women. These do not need to be large surveys but should be carefully designed to identify trends over time. Opportunities may be developed for tagging affordable sentinel STI screening onto existing data collection platforms. For example, screening and treatment for chlamydia in adolescents, as recommended in the WHO global STI strategy 2016–2021, (*29*) may yield useful data but is as yet not implemented in Mongolia. Reliable monitoring of both STIs would, furthermore, benefit from strengthening the national reporting system and expanding it to track cases treated in private-sector services, including self-treatment with drugs dispensed by pharmacies.

Our analyses confirm challenges Mongolia faces with STI case reporting; with access to laboratory facilities for diagnosis, which is largely limited to Ulaanbaatar serving just half of the national population; (*3*, *7*, *8*) and with adherence to syndrome-based case reporting. At present, Mongolia reports negligible numbers of UD cases because most providers do not follow the syndromic approach by which STI patients would get recorded by syndrome whether or not subsequently an etiological diagnosis is established. More consistent implementation of the syndromic approach for both treatment and reporting may improve completeness of treatment (avoiding loss of patients between initial syndromic diagnoses, referral to the laboratory, and waiting time for diagnosis and referral for treatment) as well as case reporting to become more usable for surveillance and planning. Additionally, reporting from pharmacies of clients presenting with UD symptoms might support surveillance. However, self-treatment with over-the-counter drugs should not be encouraged as a treatment policy due to risks of spreading antimicrobial resistance among gonococcal isolates. (*30*)

In conclusion, model-based estimations based on prevalence surveys suggest that gonorrhoea and chlamydia have declined in Mongolia but remain high. The high STI rates, much of which remains undiagnosed and untreated, bring a substantial burden of sequelae including infertility, pelvic inflammatory disease and ectopic pregnancy; they are a behavioural marker and biomedical cofactor for HIV transmission.

These results, and the wide confidence intervals around most estimates, argue for improved data input though periodic prevalence surveys beyond key populations. Our findings also highlight a largely hidden burden of untreated chlamydia that merits intensification of control efforts beyond routine clinical services. Screening should be intensified in primary care settings, among key populations, within antenatal care and for youth, e.g. via school-based clinics. New diagnostic and delivery approaches and affordable point-of-care tests (*29*) should facilitate clinic-based and non-clinic-based screening thus improving treatment coverage and surveillance and reducing disease burden.
